# Identification of a tumor microenvironment-related seven-gene signature for predicting prognosis in bladder cancer

**DOI:** 10.1186/s12885-021-08447-7

**Published:** 2021-06-10

**Authors:** Zhi Wang, Lei Tu, Minfeng Chen, Shiyu Tong

**Affiliations:** 1 Department of Urology, Hunan Children’s Hospital, No.86 Ziyuan Road, Changsha, 410007 Hunan China; 2 Department of Urology, Xiangya Hospital of Central South University, No.88 Xiangya Road, Changsha, 410008 Hunan China

**Keywords:** Bladder cancer, Microenvironment, The Cancer genome atlas, Weighted gene co-expression network analysis, Immune cell

## Abstract

**Background:**

Accumulating evidences demonstrated tumor microenvironment (TME) of bladder cancer (BLCA) may play a pivotal role in modulating tumorigenesis, progression, and alteration of biological features. Currently we aimed to establish a prognostic model based on TME-related gene expression for guiding clinical management of BLCA.

**Methods:**

We employed ESTIMATE algorithm to evaluate TME cell infiltration in BLCA. The RNA-Seq data from The Cancer Genome Atlas (TCGA) database was used to screen out differentially expressed genes (DEGs). Underlying relationship between co-expression modules and TME was investigated via Weighted gene co-expression network analysis (WGCNA). COX regression and the least absolute shrinkage and selection operator (LASSO) analysis were applied for screening prognostic hub gene and establishing a risk predictive model. BLCA specimens and adjacent tissues from patients were obtained from patients. Bladder cancer (T24, EJ-m3) and bladder uroepithelial cell line (SVHUC1) were used for genes validation. qRT-PCR was employed to validate genes mRNA level in tissues and cell lines.

**Results:**

365 BLCA samples and 19 adjacent normal samples were selected for identifying DEGs. 2141 DEGs were identified and used to construct co-expression network. Four modules (magenta, brown, yellow, purple) were regarded as TME regulatory modules through WGCNA and GO analysis. Furthermore, seven hub genes (ACAP1, ADAMTS9, TAP1, IFIT3, FBN1, FSTL1, COL6A2) were screened out to establish a risk predictive model via COX and LASSO regression. Survival analysis and ROC curve analysis indicated our predictive model had good performance on evaluating patients prognosis in different subgroup of BLCA. qRT-PCR result showed upregulation of ACAP1, IFIT3, TAP1 and downregulation of ADAMTS9, COL6A2, FSTL1,FBN1 in BLCA specimens and cell lines.

**Conclusions:**

Our study firstly integrated multiple TME-related genes to set up a risk predictive model. This model could accurately predict BLCA progression and prognosis, which offers clinical implication for risk stratification, immunotherapy drug screen and therapeutic decision.

**Supplementary Information:**

The online version contains supplementary material available at 10.1186/s12885-021-08447-7.

## Background

Bladder cancer (BLCA) is the 11th common malignancy of urinary system worldwide, and represented mainly by histological type of urothelial carcinoma [1]. Every year around 430,000 new BLCA cases are diagnosed globally with about 16,500 death cases [2]. BLCA is classified into two clinically categories at the time of diagnosis: Non-muscle invasive bladder cancer (NMIBC) and muscle invasive bladder cancer (MIBC). Although NMIBC patients usually have a good prognosis, approximately 70% of NMIBC cases will have a recurrence in the first year after initial diagnosis, and nearly 10–50% of NMIBC will progress to MIBC that is featured with higher grade and poorer prognosis [3]. Therefore, there is urgent need to highlight the BLCA carcinogenesis mechanism and find effective biomarkers to guide clinical treatment.

In recent studies, a growing body of emerging evidences raise the awareness in cancer research community that tumor microenvironment (TME) may play a pivotal role in carcinogenesis, immune evasion and treatment response [4–7]. TME is critical for both tumor maintenance and progression, as well as a desirable parameter to assess the treatment efficacy. TME is a complicated interactive network involving immune cell, stromal cell, fibroblasts, endothelial cell, blood vessels, secretory factors and extracellular matrix (ECM) [8]. Abnormal changes of extracellular environment composition and biochemical response result in disease progression at both primary site and metastatic lesion. For instance, elevated extracellular matrix metalloproteinase inducer (EMMPRIN) expression is associated with more invasive phenotype of tumor cell, advanced grade and stage in bladder cancer [9]. In addition, some studies reported that degree and ratio of tumor-infiltrating cell contributed to distinct prognosis of BLCA patients. Tumor-infiltrating neutrophils (TINs) and NLR (neutrophils-lymphocytes ratio) are significantly correlated with pathological T stage of BLCA. High TIN indicates higher risk of recurrence in NMIBC, while high Tumor-infiltrating lymphocytes (TILs) leads to longer survival [10]. Intravesical instillation of Bacille Calmette–Guérin (BCG) is standard and the most commonly-used immunotherapy for carcinoma in situ (CIS) and high-risk NMIBC following TURBT. Makito et al. [11] found that both recurrence-free survival (RFS) and progression-free survival (PFS) dramatically shortened with regulatory T cells (Treg) and tumor-associated macrophages (TAM) increasing in the tumor-enriched area after intravesical BCG therapy, implying immune cell infiltration could be an effective tool to evaluate immunotherapy response.

Therefore, strengthening our knowledge of TME and clarifying the underlying mechanism will benefit the diagnosis and treatment of BLCA. In this study, we conducted weight gene co-expression network analysis (WGCNA) and ESTIMATE algorithm to construct co-expression networks in effort to identify TME-related gene expression module. We aimed to identify hub genes in these important modules and establish a gene signature predictive model to determine risk subset of BLCA.

## Methods

### Gene expression extraction

RNA sequencing data of BLCA containing count format data were downloaded from The Cancer Genome Atlas (TCGA) database (https://www.cancer.gov/tcga). Clinical data including gender, age, grade, tumor stage and survival time were also obtained from TCGA portal. The microarray dataset GSE31684 and the corresponding clinical information data were downloaded from the Gene Expression Omnibus (GEO) database (https://www.ncbi.nlm.nih.gov/geo/), which was performed on Affymetrix Human Genome U133 Plus 2.0 Array platform. TCGA dataset was used to screen out differentially expressed genes and construct the predictive model, and GSE31684 data was applied to validate the model as an external validation dataset. The immune score, stromal score and ESTIMATE score that reflecting the TME-related cell infiltrating degree in tumor tissue of BLCA calculated using the ESTIMATE algorithm were obtained from ESTIMATE database (https://bioinformatics.mdanderson.org/estimate/). To analyze the correlation of gene expression profile and TME-related score, a total of 365 patients with completed clinical information were enrolled in this study, after filtering out samples with unknown clinical traits, lack of ESTIMATE score and invalid survival information.

### Differentially expressed genes (DEGs) screening

The “edgeR” R was employed for identifying the DEGs by R language software (version 3.5.3). Genes were excluded when too many missing values was detected or mean expression counts were less than 5. Cut-off criteria for screening DEGs were |log2fold change| ≥ 1 and false discovery rate (FDR) < 0.05.

### Constructing the gene co-expression network

The DEGs were input to construct a weighted co-expression network using R software (version 3.5.3) based on the R package “WGCNA”, as previously described [12]. Firstly, The function “goodSamplesGenes” in “WGCNA” package was applied to check if input samples and genes were qualified to build co-expression network. Secondly, Pearson’s correlation analysis of all genes was performed to construct an adjacency matrix. Then a weighted adjacency matrix were generated by a formula a_mn_ = |c_mn_|^β^ (β is a weighted parameter of the adjacency function for ensuring a scale-free network). The adjacency matrix was transformed into a topological overlap matrix (TOM). TOM could estimate the network connectivity of a gene and was used for network generation [13]. Finally, we categorized genes with similar expression profiles into the same modules, average linkage hierarchical clustering was performed according to the TOM-based dissimilarity measure with a minimum size of 40 for the genes dendrogram.

### Functional enrichment analysis

R package “clusterProfiler” was used to conduct Gene Ontology (GO) analysis for further studying the potential biological function of DEGs, including biological process, molecular function, and cellular component in R software. *P* value < 0.05 was selected as the cut-off threshold.

### Identification of hub gene and module visualization

Hub genes were defined as genes with the maximum intramodular connectivity. Firstly, the most significant module was identified. Module Membership (MM) was represented as the absolute value of Pearson’s analysis correlation between genes in order to reflect module connectivity, while Gene Significance (GS) conveyed the correlation between genes and clinical traits. In current study, Hub genes were identified according to the criteria that |MM| > 0.8 and |GS| > 0.2. All genes in respective hub module were visualized by Cytoscape to present the molecular interaction network.

### COX regression analysis

The R package “survival” was applied to perform univariate cox regression analysis for overall survival (OS) to determine the survival related genes. Genes with *p* value < 0.05 was considered as significant survival impact factor. Least absolute shrinkage and selection operator (LASSO) was conducted to further screen hub gene and construct TME related risk predictive model via “glmnet” package in R [14].

### Survival analysis and receiver operator characteristic (roc) curve analysis

Time-dependent ROC was employed to evaluate the predictive efficacy of risk score generated by our prognostic model for 1-year, 3-year and 5-year OS by using “survivalROC” package [15]. The optimal cutoff of the risk score was determined by Youden index calculation. Patients were dichotomized into high-risk group and low-risk group according to the optimal risk score cutoff of 5-year OS. Survival analysis was performed in 365 patients. The Kaplan-Meier survival curve and the log-rank test were used to estimate survival by clinical features and risk score.

### Evaluation of TME cell infiltration

TIMER (https://cistrome.shinyapps.io/timer/) is a comprehensive online database to systematically analyze immune cell infiltration across diverse cancer types. An estimated abundance of immune cells, including B cells, CD4+ T cells, CD8+ T cells, neutrophils, macrophages, and dendritic cells was performed via special statistical method with pathological approach validation [16]. TIMER was used for calculating correlation between survival-related hub genes and immune cell infiltration in this study.

### Drug sensitivity evaluation

GSCALite (http://bioinfo.life.hust.edu.cn/web/GSCALite/) is a comprehensive web-based analysis platform for gene set cancer analysis and drug sensitivity analysis. It integrated cancer genomics data of 33 cancer types from TCGA database, Drug response data from GDSC and CTRP databases as well as normal tissue data from GTEx for gene set analysis in a one-in-all data analysis workflow. In our study, we used GSCALite database to evaluate drug sensitivity of our TME-related genes in order to find out potential molecular compounds for targeted immunotherapy.

### Patients sample collection and cell lines

All ten bladder cancer specimens and adjacent normal tissues were obtained from patients during operation. The study was approved by our institutional review board, and written informed consent was provided before surgery according to the World Medical Association Declaration of Helsinki. The histology of all samples were reviewed by two independent pathologist to confirm histopathological feature. None of these patients received any surgical intervention or therapeutical medication before this study. Bladder cancer cell line T24 and immortalized uroepithelial cell line SVHUC1 were obtained from the American Type Culture Collection (ATCC). The highly invasive human bladder cancer EJ-M3 cell line was purchased from Shanghai Donghuang Biotechnology Corp. All cell lines were cultured in RPMI 1640 medium (Gibco, NY, USA) supplemented with 10% fetal bovine serum (FBS) and incubated at 37 °C in a humidified atmosphere containing 5% CO_2._

### Quantitative real-time PCR

Total RNA was extracted by using the RNeasy Mini Kit (QIAGEN, Hilden, Germany) according to the instructions. Quantitative Real-time PCR (qRT-PCR) was performed in ABI Step-One Plus PCR system (Applied Biosystems, Foster City, CA). The primers sequences for qRT-PCR was listed in Table S4.

## Results

### ESTIMATE scores and stromal scores are significantly correlated with BLCA subtypes

ESTIMATE algorithm (Estimation of Stromal and Immune cells in Malignant Tumors using Expression data) is a tool for predicting the presence of immune/stromal cells infiltration and tumor purity in tumor tissue based on single sample gene set enrichment analysis (ssGSEA). Stromal score captures the presence of stromal cells in tumor tissue, while immune score represents the infiltration of immune cells in tumor area. ESTIMATE score reflects tumor purity [17]. Gene expression profiles and clinical traits data were downloaded from TCGA database and GSE31684. Clinical information is shown in Table 1.

**Table 1:** Clinical characteristics of TCGA data and GSE31684

Datasets	TCGA	GSE31684
**Sample number**		
**Total**	365	93
**Age (years old)**		
> 70	162	41
≤ 70	203	52
**Gender**		
Male	271	68
Female	94	25
**Grade**		
High Grade	345	88
Low Grade	20	5
**Stage**		
Stage I	2	–
Stage II	101	–
Stage III	137	–
Stage IV	125	–
**T stage**		
Ta	0	8
T1	3	19
T2	116	55
T3	190	10
T4	56	1

**Fig. 1:**
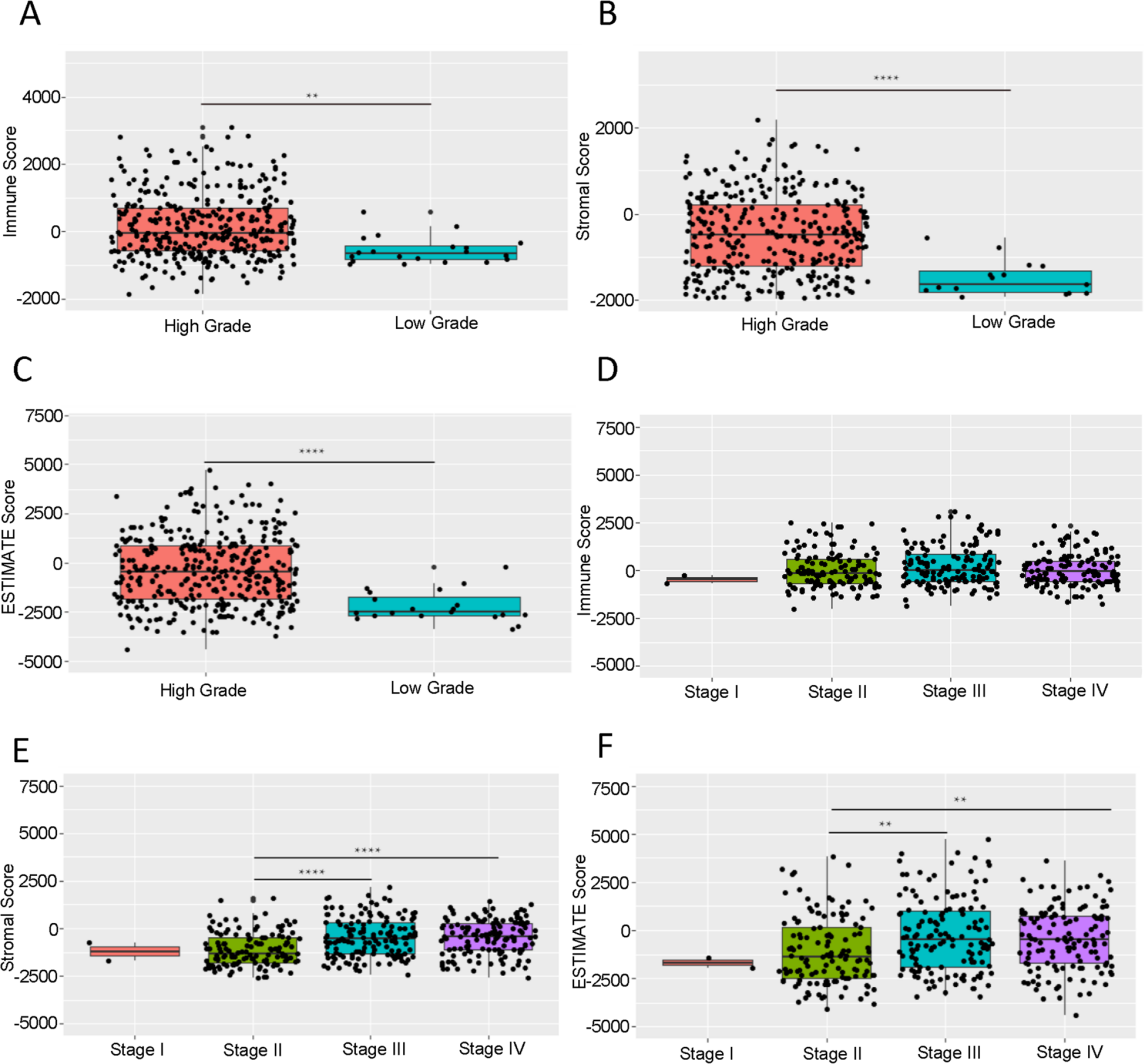
Relationship between Immune-related score and clinicopathological variables of BLCA. **A-C** Immune score, stromal score and ESTIMATE score in high- and low-grade of BLCA. **D-F** Immune score, stromal score and ESTIMATE score in different tumor stage. ** *p* < 0.01, **** *p* < 0.0001

We plotted the distribution of the immune score, stromal score and ESTIMATE score in TCGA BLCA cohort stratified by tumor grade and stage. Based on our results, the immune score ranged from − 1869.18 to 3085.28, the stromal score ranged from − 2628.68 to 2175.37, and the ESTIMATE score ranged from − 4398.47 to 4704.79. The results also showed that immune score, stromal score and ESTIMATE score were significantly higher in high grade BLCA than those in low grade BLCA (Fig. 1A-C). In addition, stromal score and ESTIAMTE score were correlated with the tumor stage, both of these two score were higher in stage III-IV compared with stage I-II. But there was no significant difference in immune score of four stages (Fig. 1D-F).

### Gene co-expression network construction of BLCA through WGCNA

After screening DEGs under the criteria of |log_2_FC| ≥ 1and *p* value < 0.05, a total of 2141 genes in 365 samples were regarded as DEGs (1083 up-regulated and 1058 down-regulated) for constructing WGCNA network (Supplementary Fig. S1A).

**Fig. 2:**
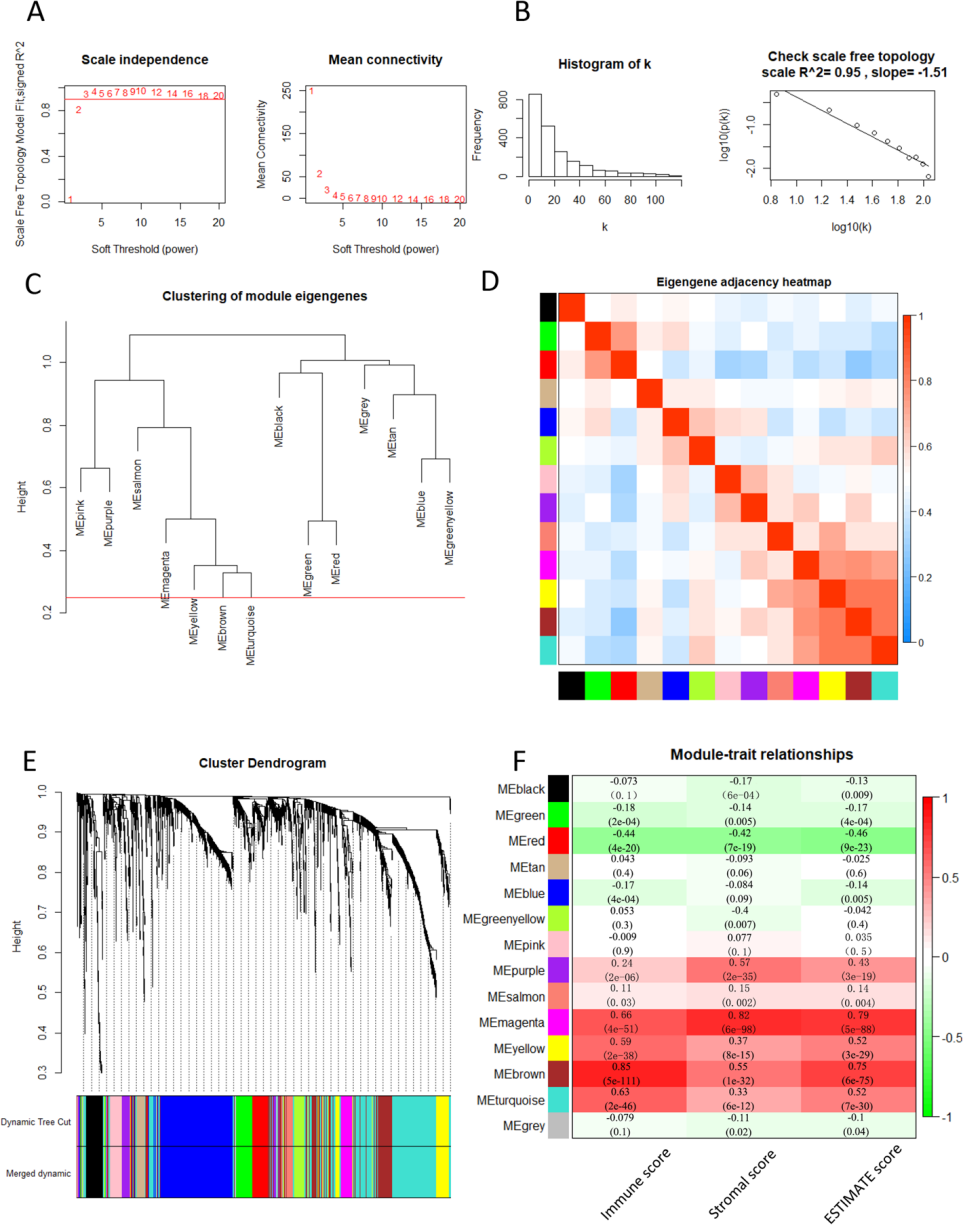
Weighted gene co-expression network of BLCA. **A** The scale-free fit index and mean connectivity for various soft-thresholding powers. **B** Histogram of connectivity distribution and Checking the scale free topology when β = 3. **C** Clustering dendrogram of consensus module eigengenes. The red line represents merging threshold is 0.25. **D** Eigengene adjacency heatmap. **E** Dendrogram of all DEGs enriched based on dissimilarity measure (1-TOM) and the corresponding cluster module colors. **F** Heatmap of the correlation between the clinical traits and module eigengenes

To ensure the reliability of the co-expression network, we employed “hclust” function to plot a clustering dendrogram for excluding outlier samples, no sample was removed from our cohort (Supplementary Fig. S1B). To build a scale-free network, β = 3 was set up as optimal soft threshold to ensure high scale independence degree (near 0.9) and low mean connectivity (close to 0) with scale free R^2^ = 0.95 (Fig. 2A-B). Module eigengenes were calculated and modules were clustered based on their correlation. Furthermore, as shown in Fig. 2C-E, 14 modules (pink, purple, salmon, magenta, black, tan, greenyellow, turquoise, yellow, blue, red, brown, green and grey module) with size ranging from 42 to 585 were identified based on their co-expression pattern, and no module needed to be merged as dissimilarity of the modules was set as 0.2. These modules contained 78 (pink), 70 (purple), 42 (salmon),75 (magenta),97 (black),46 (tan),61 (greenyellow),585 (turquoise), 142 (yellow),439 (blue),106 (red),286 (brown),106 (green) and 8 genes (grey module), respectively. Generally, genes without any significant co-expression pattern would be designated as “grey module” according to “WGCNA” algorithm, so the 8 genes in this group was removed.

To identify genes associated with BLCA TME, we analyzed the association between modules and clinical traits. The correlations between TME scores and module eigengenes were shown in Fig. 2F. This result showed that 8 modules, including red, brown, magenta, green, purple, salmon, yellow, turquoise were significantly correlated with immnue score, stromal score and ESTIMATE score. Brown module has the highest correlation with immune score (*r* = 0.85, *p* = 5e-11). In addition, ESTIMATE score (*r* = 0.79, *p* = 5e-88) were most significantly associated with magenta module by Module-feature relationship analysis. Red module was negatively correlated with all of these score (*p* < 0.05). In order to explore the function and biological relevance of genes in these modules, the lists of all genes in each module were uploaded and mapped into Gene Ontology (GO) analysis for a functional annotation analysis based on “clusterProfiler” and “org. Hs.eg.db” packages. The analysis result revealed that four out of 8 modules, magenta, brown, yellow, and purple modules were associated with TME (Supplementary Fig. S2, Supplementary Table S1).

### Identification and visualization of TME-related hub genes

**Fig. 3:**
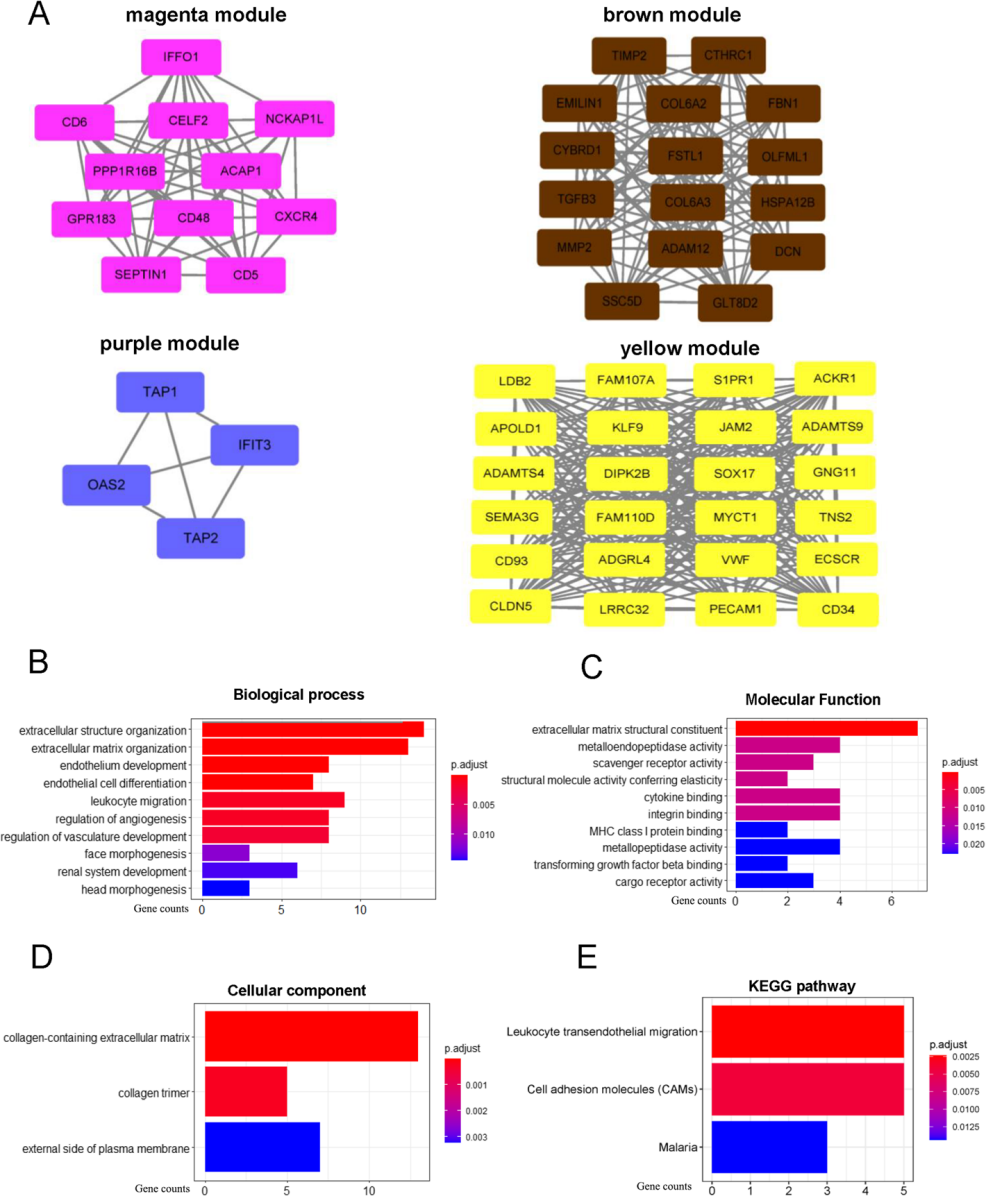
Functional enrichment analysis and PPI network of 55 hub genes. **A** Visualization of hub genes in magenta, brown, purple and yellow module, based on weight. **B** Biological process terms of GO enrichment from 55 hub genes. **C** Molecular function terms of GO enrichment from 55 hub genes. **D** Cellular component terms of GO enrichment from 55 hub genes. **E** KEGG pathway enriched from 55 hub genes

Genes with high connectivity in modules were investigated as hub genes that played a critical role in pathways coordinated with other genes. We selected 55 genes from these four key modules as hub genes, through the criterion: |Module-Membership| > 0.8, |Gene-Significance| > 0.2. There were 11, 24, 4 and 16 genes in magenta, yellow, purple and brown module, respectively. A protein-protein interaction (PPI) network was constructed and visualized using Cytoscape software, which showed these genes were highly connected in respective module (Fig. 3A, Supplementary Table S2).

To further explore the potential function of these hub genes associated with BLCA TME, GO functional and KEGG pathway enrichment analyses were conducted. In GO enrichment analysis, 55 hub genes were associated with extracellular microenvironment. A sum of 68 biological process (BP) items, 16 molecular function (MF) items, 3 cellular component items and 3 KEGG pathway were determined to significantly associated with hub genes (adjusted *p* value < 0.05, Benjamini and Hochberg method, Supplementary Table S3). BP of genes ontology analysis showed that hub genes were mainly associated with extracellular structure organization, extracellular matrix organization, endothelium development and regulation of vasculature development (Fig. 3B). MF analysis revealed that extracellular matrix structural constituent, cytokine binding, structural molecule activity conferring elasticity were predominantly involved (Fig. 3C). Collagen-containing extracellular matrix, collagen trimer and external side of plasma membrane were significantly enriched in CC analysis result (Fig. 3D). The result of KEGG pathway analysis was shown in Fig. 3E. The significantly enriched pathways were leukocyte transendothelial migration, cell adhesion molecules (CAMs) and malaria pathway.

### Construction of risk predictive model

**Fig. 4:**
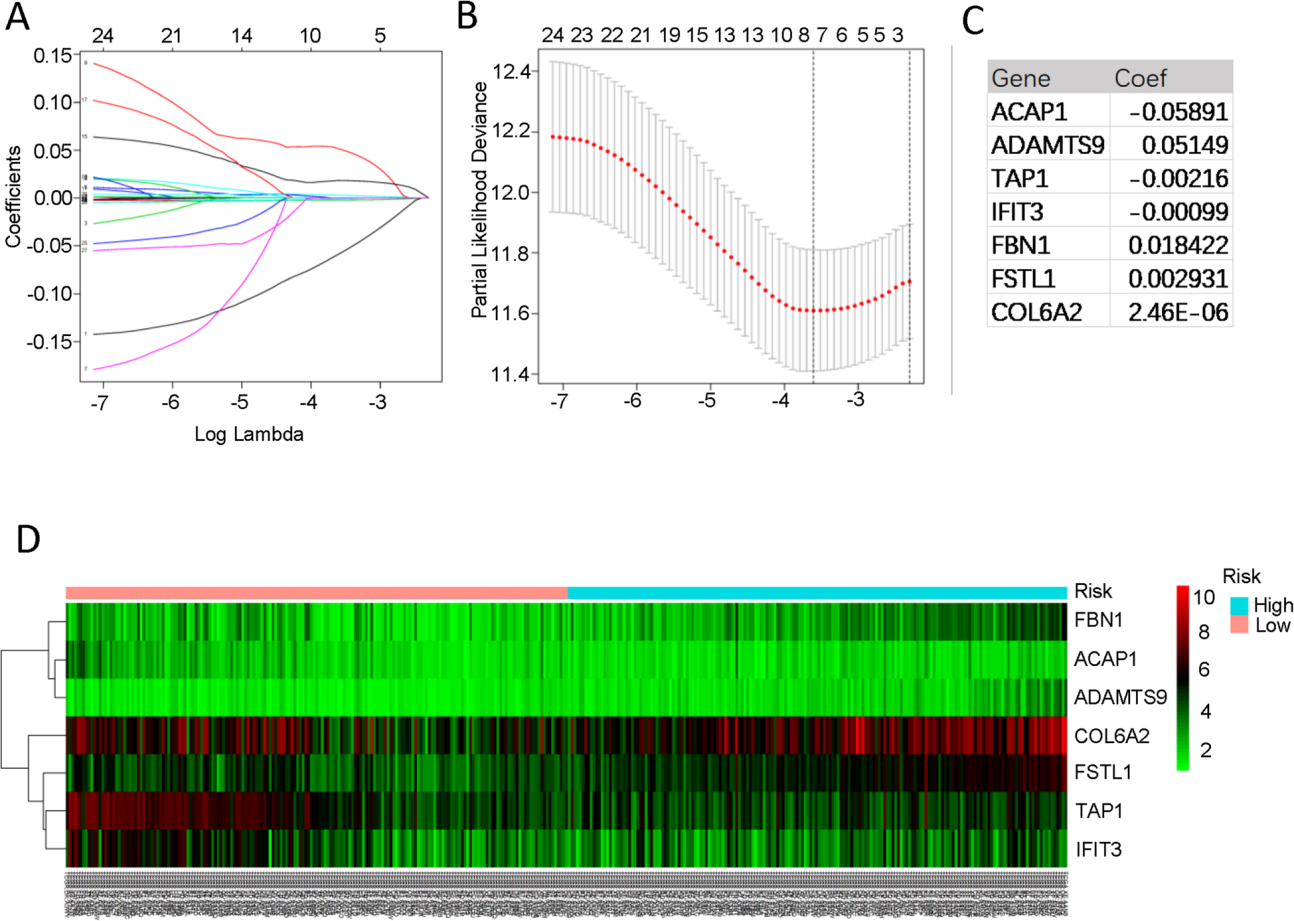
Identification of independent prognostic TME-related genes by LASSO regression. **A** LASSO coefficient profiles of 26 genes. **B** Partial likelihood deviance for the LASSO coefficient profile. **C** Seven TME-related prognostic hub genes with corresponding coefficients. **D** Heatmap of seven TME-related prognostic hub genes expression in different risk groups

**Fig. 5:**
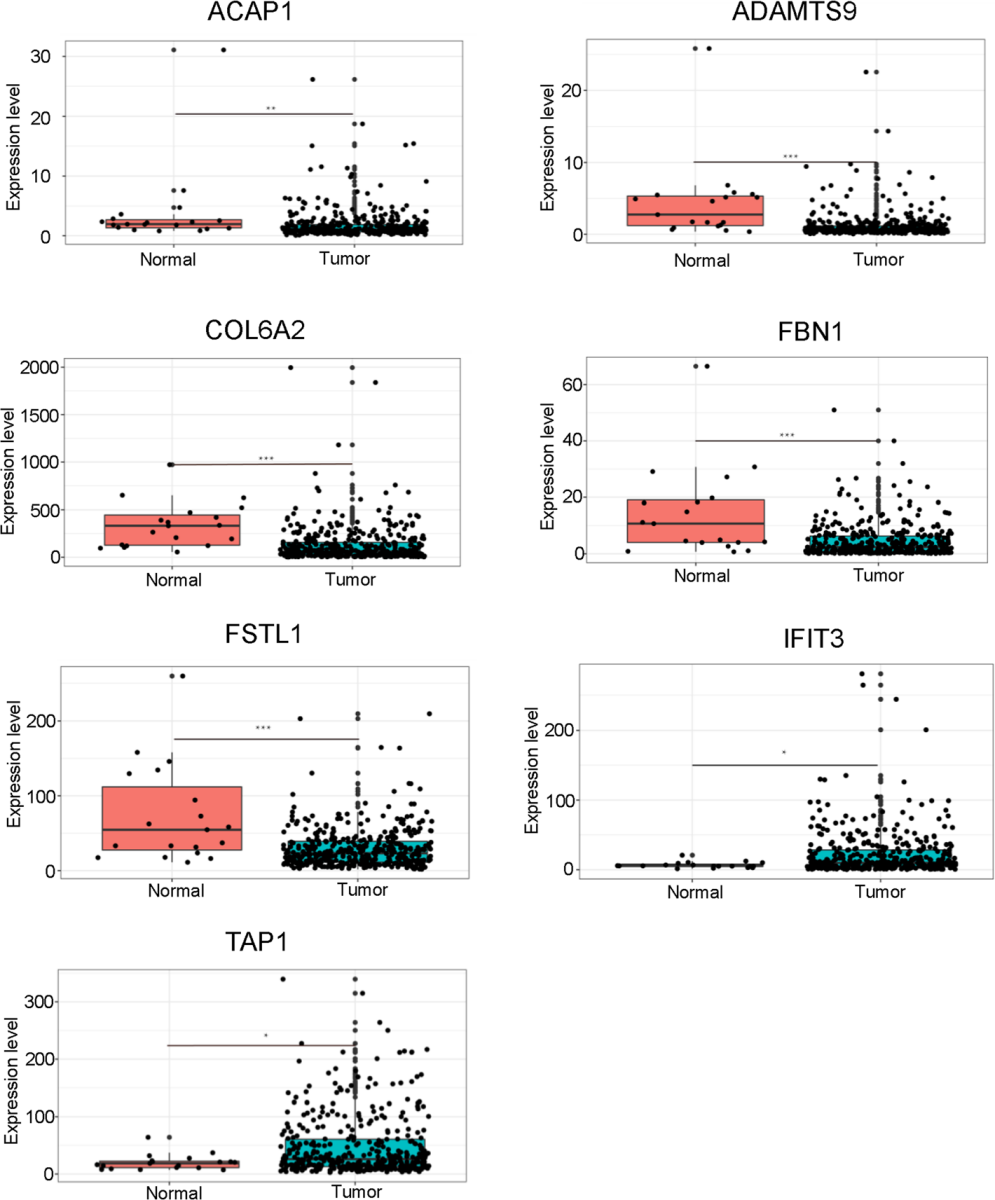
Boxplots represents expression differences of seven TME-related prognostic hub genes in tumor and adjacent normal samples. * *p* < 0.05; ** *p* < 0.01, *** *p* < 0.001

To identify the association between hub genes and BLCA prognosis, univariable Cox proportional hazards regression analysis was performed. We identified 26 out of 55 genes that were correlated to OS of BLCA in TCGA cohort. (*p* < 0.05, Table 2). In order to screen out prognostic genes for constructing risk predictive model, we conducted LASSO regression with 26 survival-related genes. LASSO regression analysis showed 7 genes were powerful prognostic factors (Fig. 4A-B). The coefficient of each factor was listed in Fig. 4C. Based on these risk predictive factors, a gene signature for risk evaluation was constructed. The risk score was calculated by the formula based on factor coefficients: Risk score = (− 0.0589 * expression value of ACAP1) + (0.0515 * expression value of ADAMTS9) + (− 0.0022 * expression value of TAP1) + (− 0.001 * expression value of IFIT3) + (0.0184 * expression value of FBN1) + (0.0029 * expression value of FSTL1) + (2.4572e-06 * expression value of COL6A2). Samples were divided into high risk and low risk group based on the cutoff of risk score median value. The heatmap showed that these prognostic genes expression were associated with risk group (Fig. 4D). The expression level of these genes was significantly different in tumor and normal tissues (*p* < 0.05, Fig.5).

**Table 2:** Univariable COX regression analysis for screening genes with prognostic value for further LASSO analysis

Gene	HR	HR.95% CI Low	HR.95% CI High	*p-*value
ACAP1	0.896998	0.807783	0.996065	0.04198
LRRC32	1.022943	1.007794	1.038319	0.002883
CD93	1.022456	1.005694	1.039496	0.008456
KLF9	1.030471	1.004251	1.057376	0.022457
GNG11	1.027446	1.005875	1.049479	0.012382
VWF	1.014997	1.003073	1.027063	0.013553
LDB2	1.106704	1.012021	1.210246	0.026295
CLDN5	1.024293	1.001453	1.047654	0.036967
ADAMTS9	1.130324	1.05282	1.213533	0.000724
TAP1	0.994936	0.991298	0.998587	0.006602
TAP2	0.975461	0.955545	0.995793	0.01825
IFIT3	0.992828	0.986062	0.99964	0.039095
OLFML1	1.099223	1.009179	1.1973	0.030044
EMILIN1	1.004574	1.000075	1.009094	0.046293
FBN1	1.036808	1.014818	1.059274	0.00095
CYBRD1	1.016109	1.001224	1.031216	0.033802
GLT8D2	1.054755	1.019277	1.091469	0.00226
TGFB3	1.028558	1.004636	1.053049	0.019015
HSPA12B	1.090216	1.01379	1.172403	0.019843
FSTL1	1.008764	1.003476	1.014079	0.001137
CTHRC1	1.003454	1.000365	1.006552	0.028366
TIMP2	1.002793	1.00043	1.005161	0.020493
COL6A3	1.004288	1.001193	1.007393	0.006586
COL6A2	1.000884	1.000268	1.001501	0.004929
ADAM12	1.029261	1.00426	1.054884	0.021516
DCN	1.004866	1.001381	1.008364	0.006177
SSC5D	1.043881	1.00232	1.087165	0.038287

**Fig. 6:**
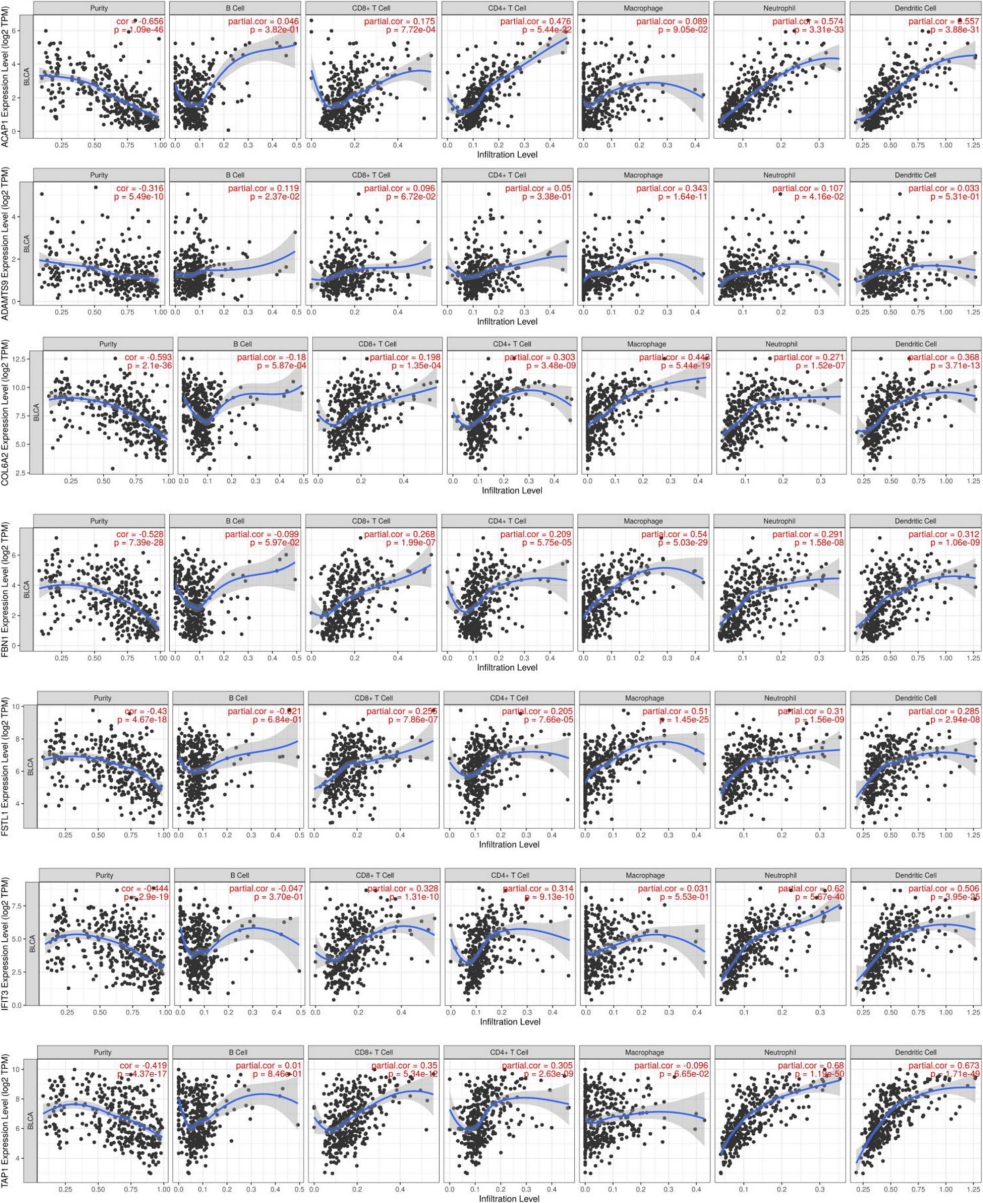
Correlation analysis between the expressions of seven TME-related prognostic hub genes and infiltration levels of B cell, CD8+ T cell, CD4+ T cell, macrophage, neutrophil, and dendritic cell

Considering potential role of prognostic genes in TME regulation and remodeling, we investigated whether our prognostic genes expression levels were correlated with the abundance of TME-related six types of immune cells in TIMER database. The result demonstrated that all these seven prognostic genes presented a variety of correlation to immune cell infiltration. As shown in Fig. 6, ACAP1 was strongly correlated with infiltration level of neutrophil (r = 0.574) and dendritic cell (r = 0.557). ADAMTS9 showed a weak correlation with infiltration level of B cell (r = 0.119) and macrophage (r = 0.343). COLA62 presented a moderate correlation with macrophage infiltration level with r value of 0.443, while FBN1 have the highest correlation with macrophage infiltration level (r = 0.54). FSTL1 was in correlation with five immune cell infiltration (r ranged from 0.255 to 0.51) except B cell. IFIT3 and TAP1 were highly correlated with infiltration level of neutrophil and dendritic cell (r ranged from 0.506 to 0.68), and relatively low correlated with other immune cells (r < 0.4).

### Prognostic value evaluation of the risk signature established based on seven TME-related genes

**Fig. 7:**
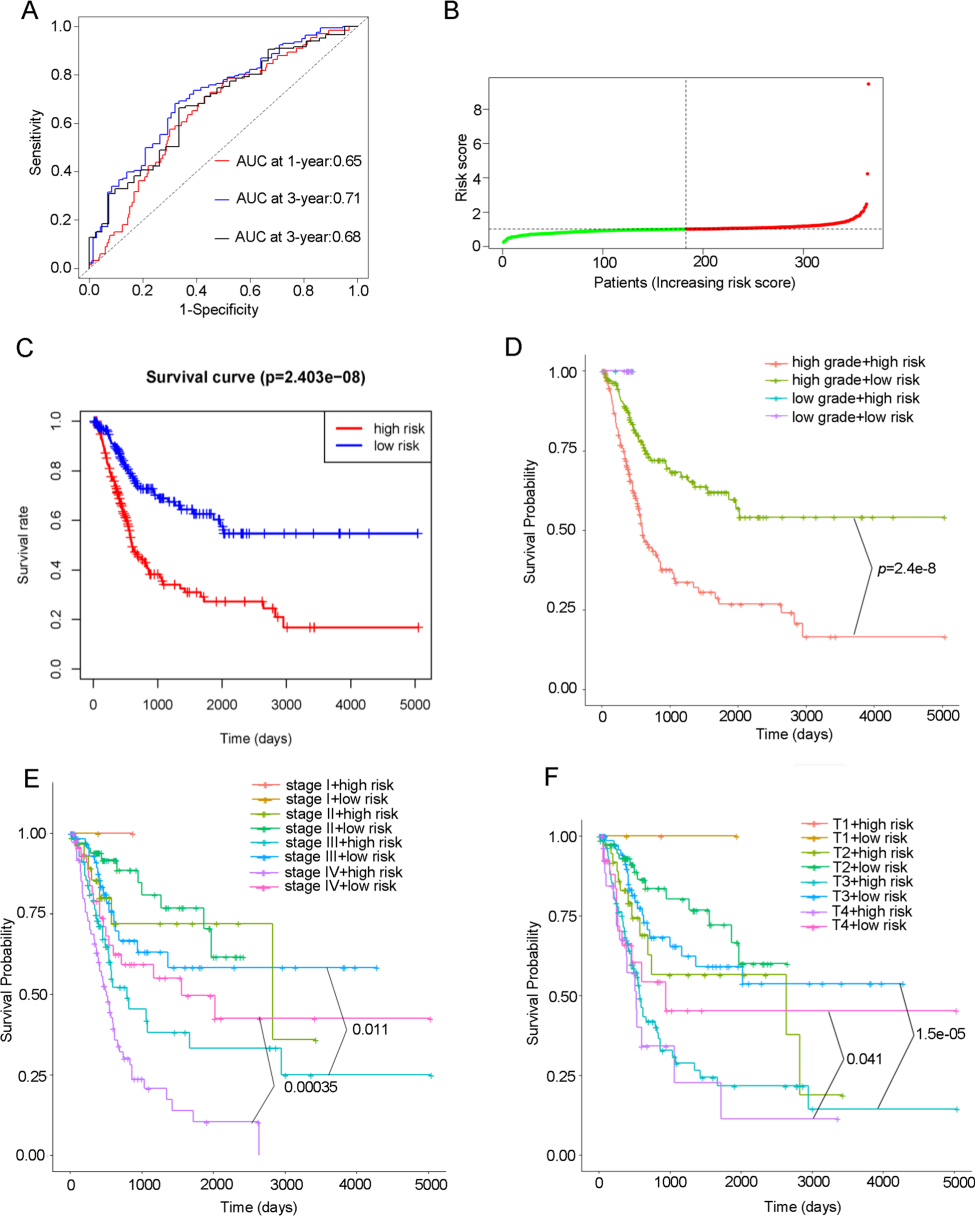
Construction of prognostic model in BLCA cohort. **A** Time-dependent ROC curve shows good performance of our risk predictive model on 1-, 3-, and 5-year OS. **B** The distribution of risk scores. **C** Kaplan-Meier curve for OS in BLCA patients stratified by the risk predictive model into high- and low-risk group. **D** Kaplan-Meier curve for OS between high- and low-grade patients with high- and low-risk. **E** Kaplan-Meier curve for OS between patients in different tumor stage with high- and low-risk. **F** Kaplan-Meier curve for OS between patients in different T stage with high- and low-risk

Time-dependent ROC curve analysis was implemented to evaluate the predictive efficacy of our prognostic model for predicting survival outcome. In our study, the Area Under Curve (AUC) of our prognostic model for 1-year, 3-year, and 5-year OS was 0.65, 0.71 and 0.68, respectively. It indicated risk scores calculated by gene signature model can accurately predict BLCA patients outcome (Fig. 7A). Patients were distributed significantly differently after divided into high-risk and low-risk group based on median value of risk score (Fig. 7B). Kaplan-Meier curve analysis was employed to assess predictive capability of risk score for OS. As shown in Fig. 7C, patients in high-risk group presented poor overall survival than those in low-risk group (*p* = 2.403e-08). We also performed stratified survival analysis to evaluated prognostic value of the risk signature in subgroups of BLCA. When stratified by tumor grade, no survival difference was observed between high- and low-risk group in low-grade subgroup, whereas low-risk group had better survival outcome than high-risk group in high-grade subgroup (Fig. 7D). When tumor stage and risk score were enrolled jointly, patients with high risk had worse prognosis in late stage (stage III and stage IV) of BLCA than those with low risk, but not in stage I/II (Fig. 7E). Similarly, poor prognosis was observed in patients with high risk in T3-T4 subgroup when patients were stratified by T stage (Fig. 7F).

**Fig. 8:**
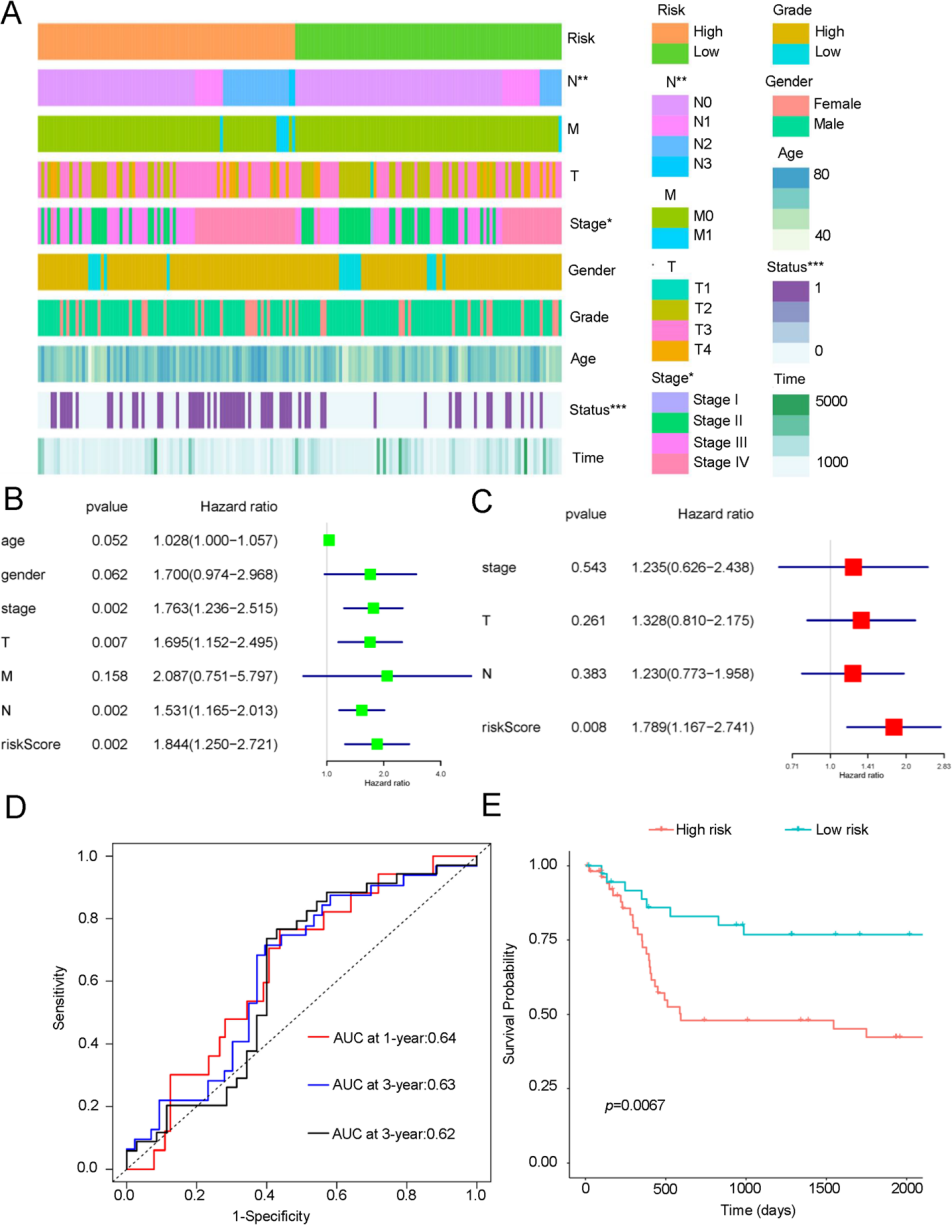
Relationship between the seven-gene signature performance and clinicopathological variables. **A** Heatmap of the distribution of clinicopathological variables between the high- and low-risk groups. **B** Univariable COX regression analysis of clinicopathological variables and risk score for OS. **C** Multivariable COX regression analysis of clinicopathological variables and risk score for OS. **D** Time-dependent ROC validates model predictive efficiency in GSE31684. **E** Kaplan-Meier curve for OS in GSE31684 stratified by the risk predictive model into high- and low-risk group. Status: Survival status, Time: Survival time * *p* < 0.05; *** *p* < 0.001

To identify the association between risk group and BLCA clinicopathological factors, we calculated the statistical difference of these factors in high-risk and low-risk groups through chi-square test. The heatmap showed that significant differences between the high-risk and low-risk group in N stage, tumor stage and survival status (Fig. 8A). We then conducted univariate and multivariate Cox regression analyses to determine whether the risk signature is an independent prognostic factor. In univariate analysis result, the risk score, T stage, N stage and tumor stage were significantly correlated with the OS (*p* < 0.05). When enrolling these variables into the multivariate Cox regression analysis, the risk score remained significantly associated with the OS (*p* < 0.05, Fig. 8B-C). Furthermore, this model was validated in external validation dataset GSE31684. In GSE31684, the AUC of this model for 1-, 3-, and 5-year OS was 0.64, 0.63 and 0.62, respectively (Fig. 8D). Kaplan-Meier also showed significantly different OS in high- and low-risk group of GSE31684 cohort (Fig. 8E). These results confirmed that the risk signature derived from TME-related genes is a risk factor for BLCA and can predict BLCA prognosis as an independent factor.

### Drug sensitivity analysis

In drug sensitivity evaluation, we analyzed the correlation between TME-related genes expression and small molecular compounds IC50 in Genomics of Drug Sensitivity in Cancer (GDSC) database via GSCALite platform. We found low expression of ACAP1 was sensitive to over 70 small molecular drugs, including IKK inhibitor TPCA-1, ATM kinase inhibition CP466722 and Hsp90 inhibitor SNX-2112. High expression of FSTL1 contributed to desirable drug response to histone deacetylases (HDAC) inhibitor vorinostat, CDK inhibitor AT7519 and cytotoxic drug methotrexate (Supplementary Fig. S3).

#### TME-related gene validation in vitro

**Fig. 9:**
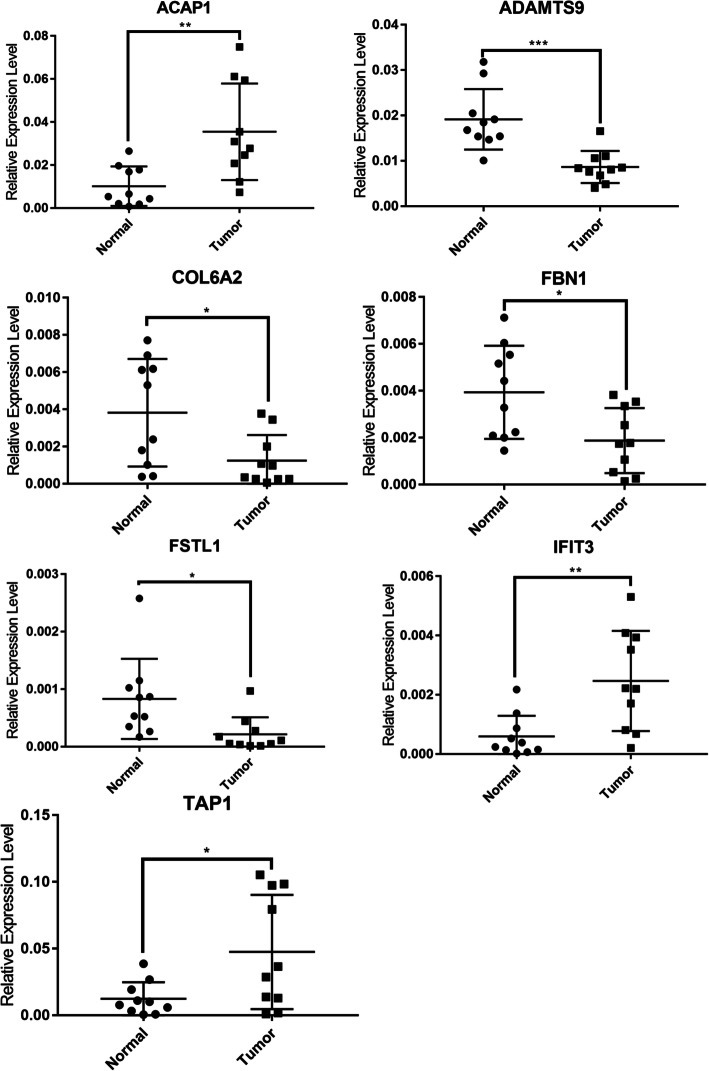
Gene validation in clinical specimens by qRT-PCR. * *p* < 0.05; ** *p* < 0.01, *** *p* < 0.001

**Fig. 10:**
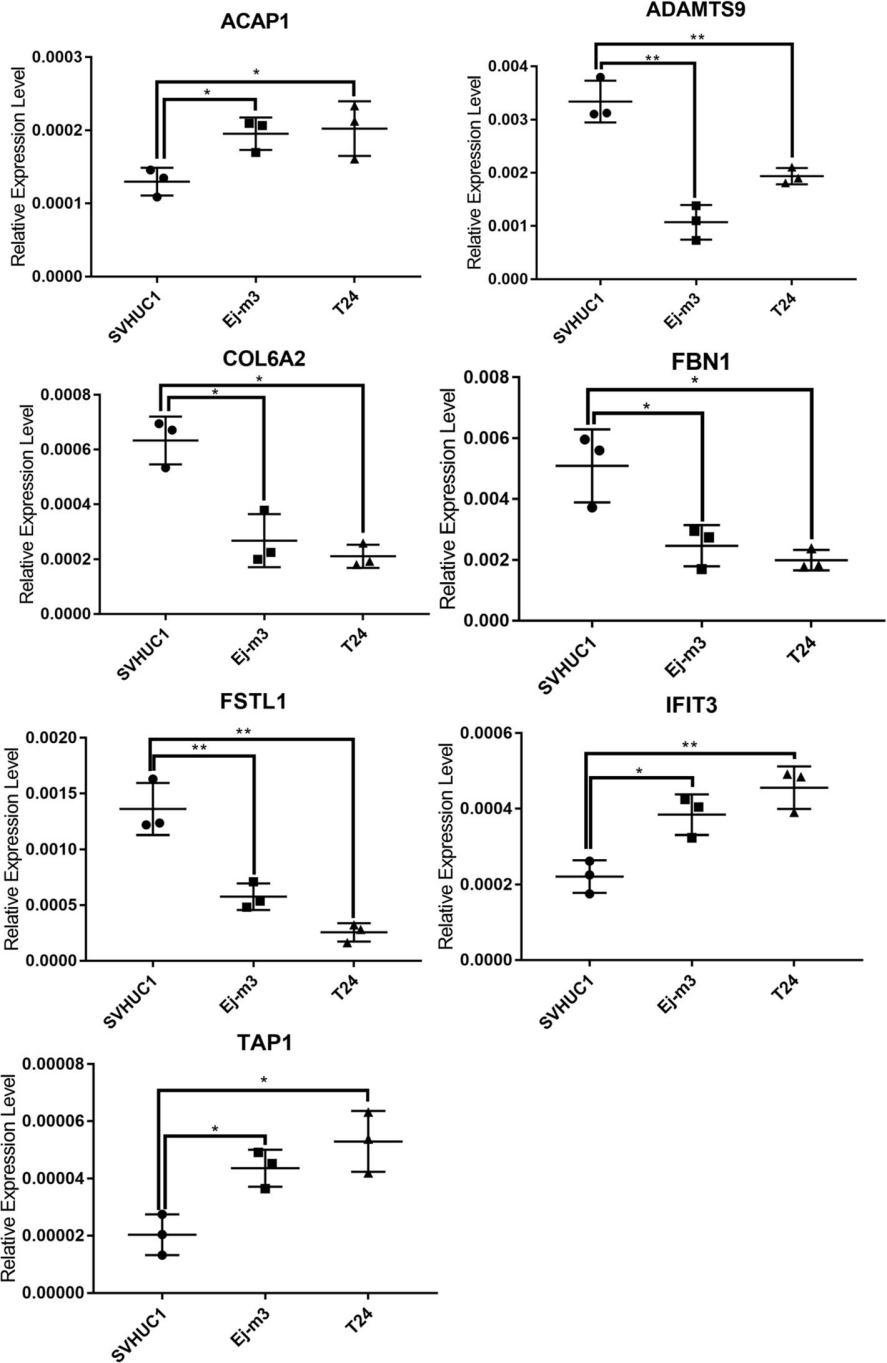
Gene validation in cell lines by qRT-PCR. * *p* < 0.05; ** *p* < 0.01

To validate these TME-related genes expression in BLCA patients, we collected 10 BLCA clinical specimens and adjacent normal tissue and performed RT-PCR. We found ACAP1, IFIT3 and TAP1 were upregulated in tumor samples, while ADAMTS9, COL6A2, FBN1 and FSTL1 were downregulated in tumor specimens (Fig. 9). To further validate it in vitro, bladder cancer (T24, EJ-M3) and bladder uroepithelial cell line (SVHUC1) were employed in gene expression validation. We found that these seven TME-related genes were also deregulated in T24 and EJ-M3 cell line (Fig. 10).

## Discussion

In recent years, a growing body of studies have demonstrated that tumor microenvironment plays a pivotal roles in tumorigenesis and response to treatments [18, 19]. Moreover, Prognostic gene signature based on TME-related geneswas been investigated in a wide range of cancers, including glioblastoma, lung carcinoma and hepatocellular carcinoma [20–22]. However, the expression profile-based risk signature in BLCA has not been established. Previous studies simply focused on expression value of multiple genes to stratify patients and predict the outcome [23, 24], which lack of rigorous accuracy for comprehensive assessment of BLCA prognosis. Therefore, in order to establish a predictive model with prognostic value, we mined transcriptome data of 365 tumor and 19 adjacent normal samples from TCGA database and eventually identified seven-gene signature by analyzing immune features and gene expression profile jointly.

ESTIMATE analysis can infer tumor cellularity and various TME cell infiltration via evaluating based on distinct properties of the gene transcriptional expression profiles of cancer samples. This bioinformatics algorithm has been widely used in tumor immune research, including in glioblastoma, hepatocellular carcinoma, acute myeloid leukemia and breast cancer [21, 22, 25, 26]. In our study, we included 365 tumor samples and 19 adjacent normal tissue with completed clinical information from TCGA database . Through ESTIMATE algorithm, we observed immune score, stromal score and ESTIMATE score were positively correlated with tumor grade. High grade tumor had higher score than low grade BLCA. Stromal and ESTIMATE score, two generally accepted indicator for poor prognosis in recent publications, increased with tumor stage (Fig. 1). European Association of Urology Guidelines 2020 gave the definition of locally advanced bladder cancer: T3-T4, N0/N1, M0, which were linked to a more aggressive phenotype with extravesical invasion [27]. Tumor progression is usually accompanied by specific cellular response or molecular alteration in TME. In invasive and metastatic tumor, intercellular bonds are loosened when individual cancer cells or a group of cancer cells leave the carcinoma in situ to disseminate [28]. Mast cells in tumor microenvironment were reported to strengthen bladder cancer metastasis by regulating ERβ/CCL2/CCR2 EMT/MMP9 signals [29]. IL-6 and STAT3 in TME are linked with angiogenesis that is the step for tumor migration and metastasis [30]. Taken together, the advanced tumor is usually featured with more noteworthy changes in TME structure, regulators or infiltration cells. This observation is consistent with TME regulation theory implied from previous studies, suggesting that bladder cancer tumorigenesis and progression is potentially associated with immune cell infiltration. Thus, we hypothesize that non-tumor constituent TME cell infiltration might participate in BLCA progression.

Considering similarities of all genes across samples, WGCNA provides a systematic approach to explore the correlation and function of the whole transcriptome. WGCNA algorithm weighs on genes regulatory role in molecular pathway and biological process rather than simply gene expression levels [31]. After applying WGCNA algorithm, 14 co-expression modules were identified via the dynamic tree cut method (Fig. 2). Then we screened out eight co-expression modules that were correlated with immune score, stromal score, and ESTIMATE score. Furthermore, we obtained four modules with a sum of 573 genes correlated to innate immune response, such as cytokine activity and leukocyte migration. Of note, extracellular structure organization were concurrently enriched in multiple modules, indicating that extracellular structure organization is one of the major target for BLCA microenvironment abnormal regulation (Supplementary Fig. S2). A cluster of genes with highest internal connectivity and clinical feature correlation are defined as hub genes, which are usually located at central position in network [32]. 55 hub genes were screened out under a rigid connectivity evaluation criteria. By function analysis, we found hub genes from these four modules were significantly enriched in the immune response and extracullar structure formation. KEGG pathway analysis revealed hub genes were enriched in leukocyte transendothelial migration and cell adhesion (Fig. 3). This finding supports the hypothesis that hub genes might play key role in BLCA tumorigenesis and progression by altering immune response pattern and microenvironment structure, which contributed to the poor prognosis of BLCA.

Considering that previous studies have only focused on expression value of multiple genes to stratify patients and predict the outcome [23, 24], we attempted to develop a gene-signature formula model. LASSO is a widely used approach for establishing prognostic gene signature models, of which main advantage is the ability of preventing overfitting [33] .Based on the 55 genes, we identified seven TME related hub genes that could be enrolled in construction of predictive model via univariate COX and LASSO regression analysis (Fig. 4). In addition, we conducted RT-PCR to quantify the expression level of these 7 genes. This in vitro assay turned out a similar trend in clinical specimens and well-established cell line, which provided vigorous evidence to support the importance and functional role of these genes in tumor environment modulation.

Increasing numbers of studies demonstrated infiltration of immune cells has a prognostic prediction value for clinical management of BCLA patients [34–36]. In our study, ADAMTS9, COL6A2, FBN1 and FSTL1 showed significant correlation with infiltration level of macrophage, while ACAP1, IFIT3 and TAP1 presented a strong correlation with infiltration level of neutrophil and dendritic cell. Other immune cells also exhibited some novel correlations to these hub genes, which had never been reported before. In some published studies, ACAP1 was found to be associated with tumor purity and CD8+ T cell toxicity in BCLA basal squamous subtype and luminal infiltrated subtypes [37]. ADAMTS9, a disintegrin and metalloproteinase with a thrombospondin type-1 motif, could be induced in fibroblasts following CD4+ T cell co-culture, leading to change of ECM components [38]. FSTL1 was found to strengthen the antigen presentation ability of drendritic cell by activating NF-κb pathway in nasopharyngeal carcinoma [39]. Thus, our finding does not only lay a potential foundation of participation of these gene in BLCA TME, but also provided new evidences to the interaction between gene alteration and immune cell infiltration.

Thus, based on the 7 genes, we established a seven-gene signature model for predicting prognosis of patients. We firstly classified patients by the risk score of each patient. Our result demonstrated that the seven-gene-based classifier had the ability to distinguish the high-risk group patients from those in the low-risk group effectively. Correlation analysis showed patients in these two groups had distinct tumor stage, N stage and prognostic consequenc. Furthermore, after adjusted for clinicopathological factors, the risk score was not only an prognostic factor independent of tumor stage, but also associated with overall survival. We further validated this signature by performing survival analysis and ROC analysis based on another independent datasets GSE31684. The result was consistent with our findings in TCGA data, which made our result more convincing. Taken together, this gene signature model was capable of distinguish high-risk and low-risk group well and have great predictive value of prognosis of BLCA patients.

TPCA-1 is a small molecular compound targeting inhibitory κB (IκB) kinase complex in NF-κB pathway. It was reported to block TNFα-induced IL-8 secretion to inhibit head and neck squamous cell carcinoma progression and enhance sensitivity to TNFα-induced cell death [40]. SNX-2112 remarkably enhanced TRAIL-induced cytotoxicity, promoted the accumulation of reactive oxygen species (ROS) and disrupted Akt/mTOR signaling pathway in cervical cancer cells [41]. CDK inhibitor AT7519 could suppress phosphorylation of CDK1, CDK2 and RNA polymerase II in colon and cervical cancer cells as well as overcome chemoresistance [42]. But few studies focused on usage and assessment of these compounds in BLCA. Our study found TME-related genes may be new potential biomarkers for target selection of these drugs. We found a strong correlation between TME-related genes and drug sensitivity, which is very helpful for future research on the individualized therapy in BLCA.

There are also several limitations in our study. Firstly, the limited size of samples and the nature of retrospective cohort study might compromise its validity. Secondly, our study lacks enough validation experiments in vitro and vivo to elucidate the molecular mechanism of these genes regulation.

In summary, using weighted gene co-expression analysis, our study identified seven key genes associated with tumor microenvironment. A risk signature with these seven genes can independently predict the prognosis of BLCA patients. These TME-related genes were aberrantly expressed and remarkably associated with immune cell infiltration in BLCA, implying that their potential role of targets for immunotherapy.

## Supplementary Information

**Additional file 1: Table S1**. GO analysis of 4 TME-related co-expression modules (magenta, brown, purple and yellow).

**Additional file 2: Table S2**. Protein-Protein interaction network information.

**Additional file 3: Table S3**. GO and KEGG pathway enrichment analysis of 55 hub genes.

**Additional file 4: Fig. S1**. WGCNA analysis of differentially expressed genes. (A) The volcano plot of differentially expressed genes. Red dots represent overexpression genes, green dots represent low expression genes, and black dots represent genes without significantly differential expression. (B) Sample dendrogram and clinical traits. The clustering was based on Pearson correlation coefficients between samples. The color intensity was proportional to immune score, stromal score and ESTIMATE score of tumor samples.

**Additional file 5: Fig. S2**. GO enrichment analysis for four TME-related modules. (A) Biological process terms of GO enrichment analysis of genes in magenta module. (B) Molecular function terms of GO enrichment analysis of genes in magenta module. (C) Biological process terms of GO enrichment analysis of genes in yellow module. (D) Molecular function terms of GO enrichment analysis of genes in yellow module. (E) Biological process terms of GO enrichment analysis of genes in brown module. (F) Molecular function terms of GO enrichment analysis of genes in brown module. (G) Biological process terms of GO enrichment analysis of genes in purple module. (H) Molecular function terms of GO enrichment analysis of genes in purple module.

**Additional file 6: Fig. S3**. The drug resistance analysis of seven TME-related genes based on GDSC IC50 drug data. Spearman correlation represents drug response to input genes. The positive correlation means that the gene high expression is resistant to the drug, vise verse.

## Data Availability

Publicly available datasets were analyzed in this study. The datasets analyzed during the current study are available in the repository below: https://portal.gdc.cancer.gov; https://bioinformatics.mdanderson.org/estimate/; https://cistrome.shinyapps.io/timer/; https://www.ncbi.nlm.nih.gov/geo/query/acc.cgi?acc=GSE31684. All data used and/or analyzed during the current study are available from the corresponding authors upon reasonable request.
