# Supplementary figures and images for: Identification of a tumor microenvironment-related seven-gene signature for predicting prognosis in bladder cancer
Source: BMC Cancer. 2021 Jun 10;21:692. doi: 10.1186/s12885-021-08447-7 (PMC8194149; doi:10.1186/s12885-021-08447-7)

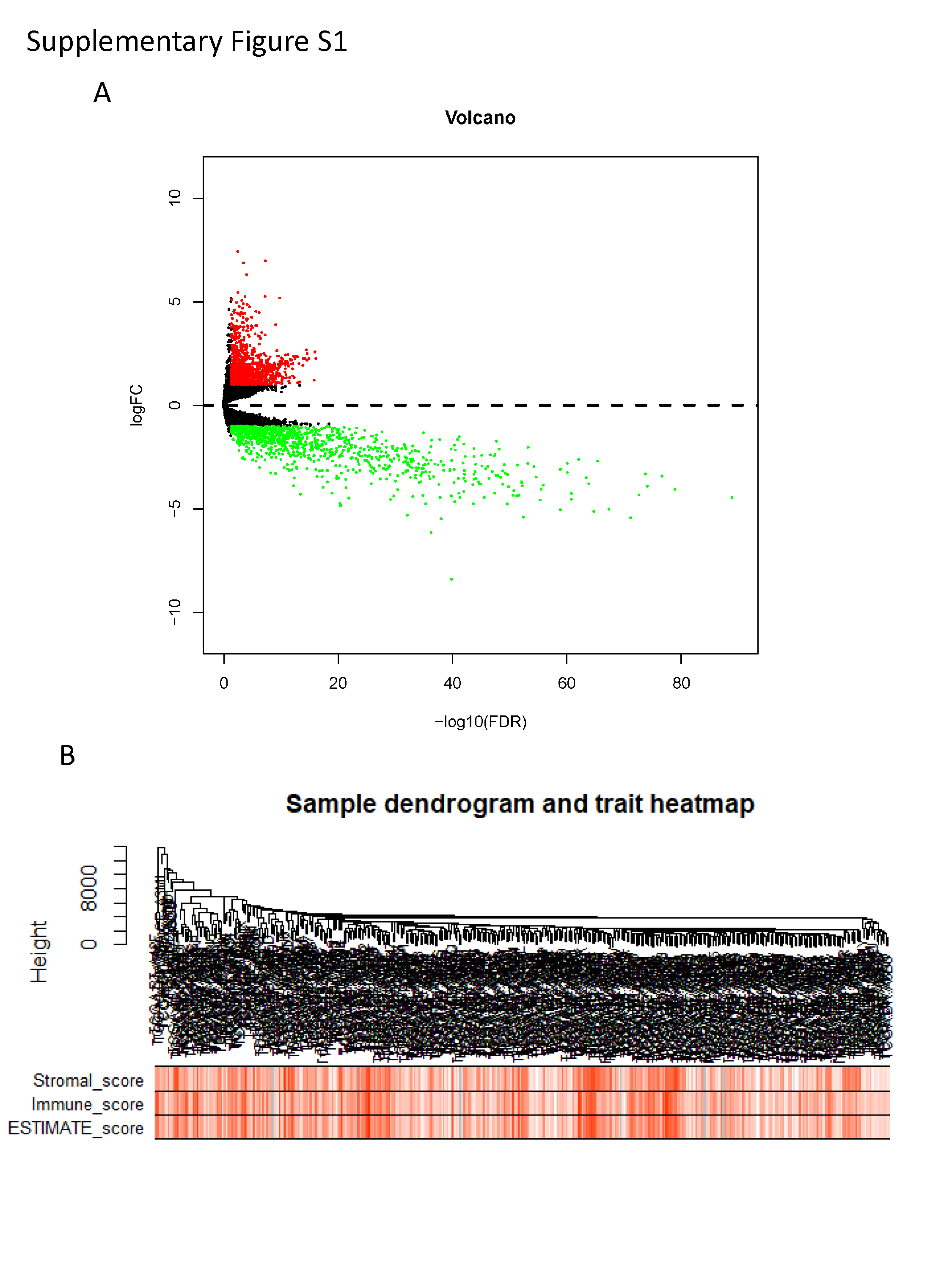

Supplement: Supplementary file 4 — Additional file 4: Fig. S1. WGCNA analysis of differentially expressed genes. (A) The volcano plot of differentially expressed genes. Red dots represent overexpression genes, green dots represent low expression genes, and black dots represent genes without significantly differential expression. (B) Sample dendrogram and clinical traits. The clustering was based on Pearson correlation coefficients between samples. The color intensity was proportional to immune score, stromal score and ESTIMATE score of tumor samples. [file 12885_2021_8447_MOESM4_ESM.tiff]

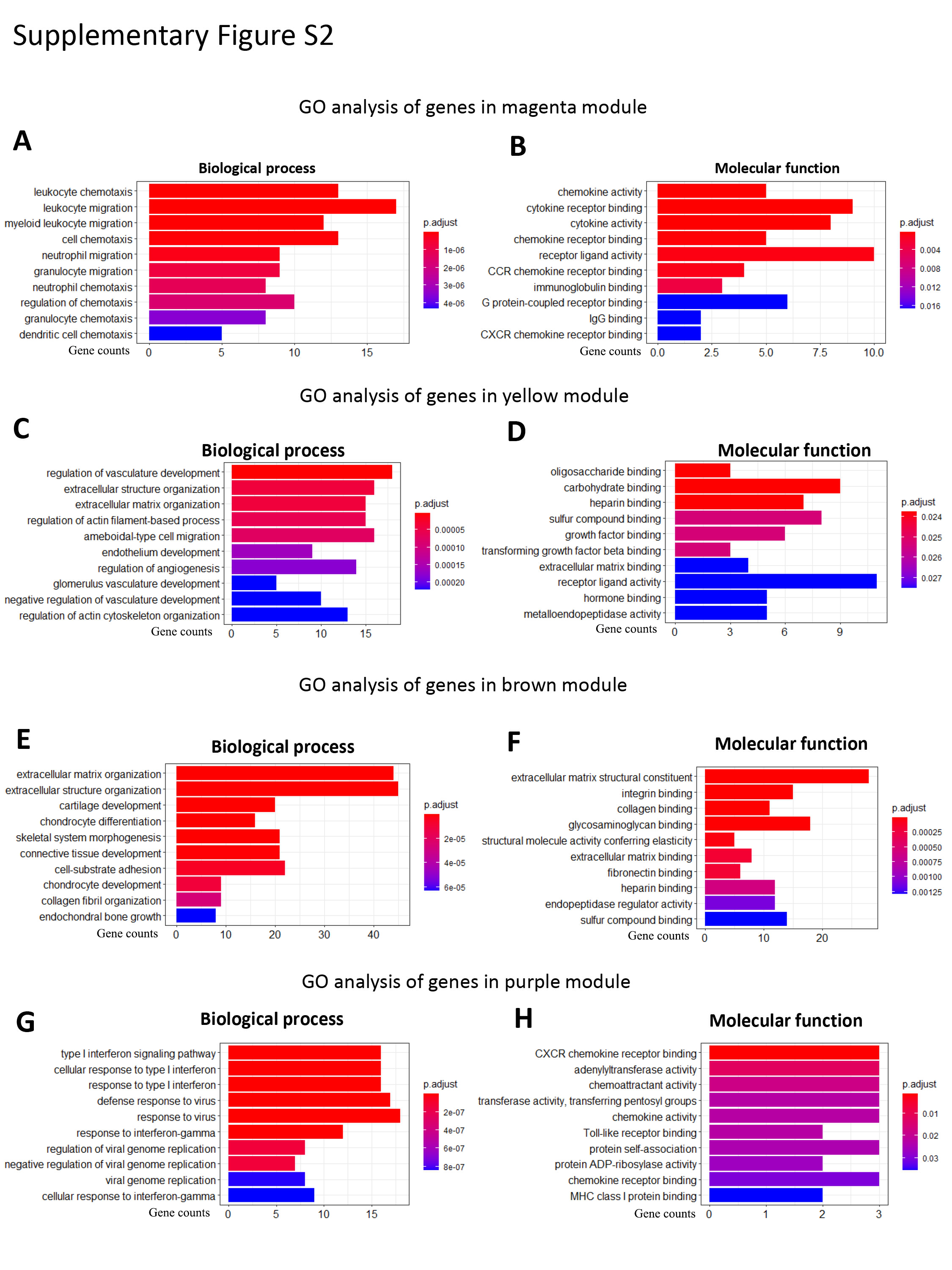

Supplement: Supplementary file 5 — Additional file 5: Fig. S2. GO enrichment analysis for four TME-related modules. (A) Biological process terms of GO enrichment analysis of genes in magenta module. (B) Molecular function terms of GO enrichment analysis of genes in magenta module. (C) Biological process terms of GO enrichment analysis of genes in yellow module. (D) Molecular function terms of GO enrichment analysis of genes in yellow module. (E) Biological process terms of GO enrichment analysis of genes in brown module. (F) Molecular function terms of GO enrichment analysis of genes in brown module. (G) Biological process terms of GO enrichment analysis of genes in purple module. (H) Molecular function terms of GO enrichment analysis of genes in purple module. [file 12885_2021_8447_MOESM5_ESM.tif]

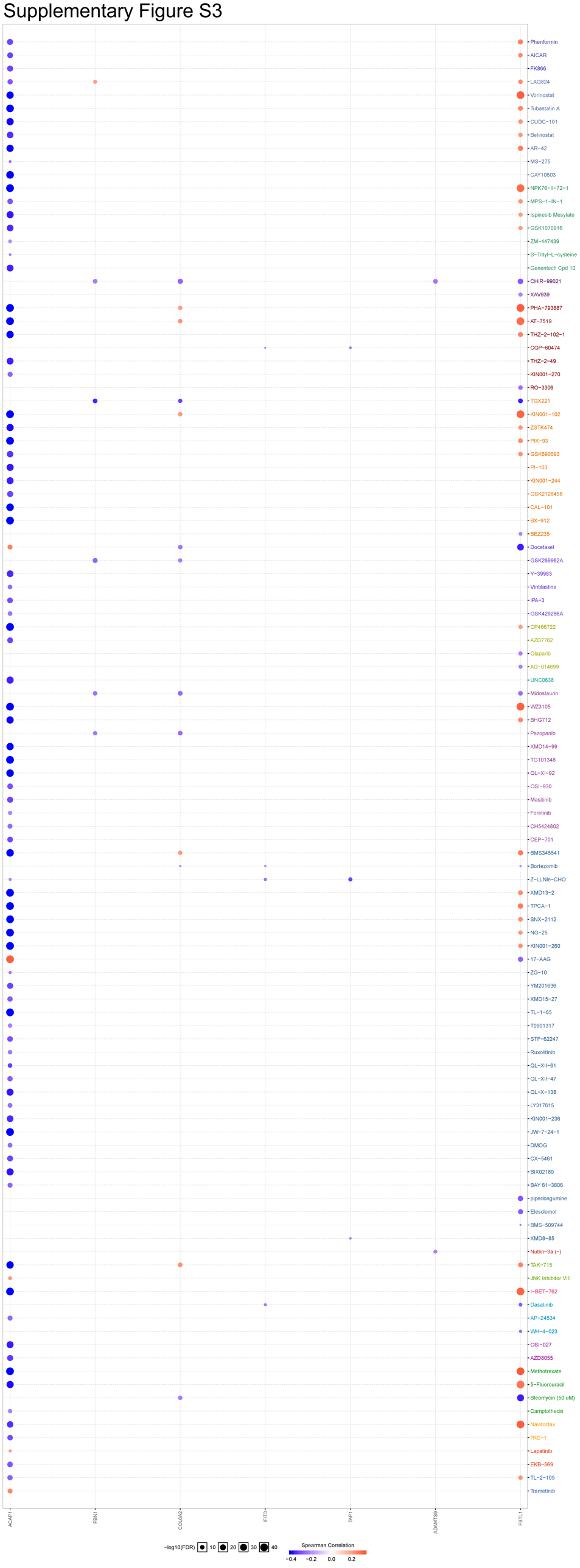

Supplement: Supplementary file 6 — Additional file 6: Fig. S3. The drug resistance analysis of seven TME-related genes based on GDSC IC50 drug data. Spearman correlation represents drug response to input genes. The positive correlation means that the gene high expression is resistant to the drug, vise verse. [file 12885_2021_8447_MOESM6_ESM.tif]
